# Contagious Furunculoid Disease
*On the Pathology and Treatment of the Contagious Furunculoid Disease. By Thomas Laycock, M.D., Professor of Medicine in the University of Edinburgh. Edinburgh: 1856.


**Published:** 1857-01

**Authors:** 


### THE EPITOME OF SANITARY LITERATURE.

### CONTAGIOUS FURUNCULOID DISEASE.*

UNDER the head of Contagious Furunculoid, Dr. Laycock includes eight forms of disease developed on the skin; viz., the simple furuncle; effusive inflammation of the derma; suppurative inflammation of the derma; carbuncular inflammation; two or more of these varieties occurring together; sloughing gangrene of the lip, eye, and tongue; diffused inflammation of the cellular tissue (phlegmon); and whitlow.

Dr. Laycock believes in the contagious character of boils, and quotes a case from a paper of Dr. Richardson's, in which the disease seemed to have propagated from a mother *enceinte* to the unborn child. In some cases the contagion may spread from the inferior animals to man, as from the horse. In this case, the form of disease is to be considered as special. The recent boil epidemic has been generally prevalent throughout the world. In England and America it has been coincident with various epidemics, as typhus, influenza, cholera, small-pox, scarlatina, hooping-cough, and croup. This coincidence is probably no more than an "epiphenomenon". The treatment is to improve the general health, and to render the local mischief abortive by the application of iodine tincture or caustic. It remains an important question, whether the eating of diseased animal food may not be one means by which the disease is communicated.

* On the Pathology and Treatment of the Contagious Furunculoid Disease. By THOMAS LAYCOCK, M.D., Professor of Medicine in the University of Edinburgh. Edinburgh: 1856.

